# Increased S100A15 expression and decreased DNA methylation of its gene promoter are involved in high metastasis potential and poor outcome of lung adenocarcinoma

**DOI:** 10.18632/oncotarget.17391

**Published:** 2017-04-24

**Authors:** Yung-Che Chen, Meng-Chih Lin, Chang-Chun Hsiao, Yi-Xin Zheng, Kuang-Den Chen, Ming-Tse Sung, Chung-Jen Chen, Ting-Ya Wang, Yong-Yong Lin, Huang-Chih Chang, Yu-Mu Chen, Jen-Chieh Chang

**Affiliations:** ^1^ Division of Pulmonary and Critical Care Medicine, Kaohsiung Chang Gung Memorial Hospital and Chang Gung University College of Medicine, Kaohsiung, Taiwan; ^2^ Center of Translational Research in Biomedical Sciences, Kaohsiung Chang Gung Memorial Hospital and Chang Gung University College of Medicine, Kaohsiung, Taiwan; ^3^ Department of Pathology, Kaohsiung Chang Gung Memorial Hospital and Chang Gung University College of Medicine, Kaohsiung, Taiwan; ^4^ Division of Rheumatology, Kaohsiung Chang Gung Memorial Hospital and Chang Gung University College of Medicine, Kaohsiung, Taiwan; ^5^ Department of Medical Research, Kaohsiung Chang Gung Memorial Hospital and Chang Gung University College of Medicine, Kaohsiung, Taiwan; ^6^ Graduate Institute of Clinical Medical Sciences, College of Medicine, Chang Gung University, Kaohsiung Chang Gung Memorial Hospital, Kaohsiung, Taiwan; ^7^ Center for Shockwave Medicine and Tissue Engineering, Kaohsiung Chang Gung Memorial Hospital, Kaohsiung, Taiwan

**Keywords:** lung adenocarcinoma, S100A15, DNA methylation, next generation sequencing

## Abstract

**Purpose:**

This study aims to determine the functional role of S100A15 and its promoter DNA methylation patterns in lung cancer progression.

**Experimental Design:**

We analyzed 178 formalin-fixed paraffin embedded specimens from lung cancer patients, including 24 early stage and 91 advanced stage adenocarcinoma. S100A15 protein expression was evaluated by immunohistochemistry stain, and its DNA methylation levels were measured by pyrosequencing.

**Results:**

S100A15 nuclear staining was increased in lung adenocarcinoma patients with distant metastasis versus those without distant metastasis. There was reduced one/three-year overall survival in adenocarcinoma patients receiving first line target therapy and harboring high nuclear expressions of S100A15. Both DNA methylation levels over -423 and -248 CpG sites of the S100A15 gene promoter were decreased in adenocarcinoma patients with distant metastasis, and the former was associated with lower one-year overall survival. The highly invasive CL1-5 cell lines display decreased DNA methylation over −412/−248/−56 CpG sites of the S100A15 gene promoter and increased S100A15 gene/protein expressions as compared with the less invasive CL1-0 cell lines. Knockdown of S100A15 in CL1-5 cell line inhibited cell proliferation, migration, and invasion, while over-expression of S100A15 in CL1-0 cell line promoted cell proliferation, migration, and invasion. RNA sequencing analysis revealed potential biological effects of S100A15 over-expression and knock-down with CTNNB1, ZEB1, CDC42, HSP90AA1, BST2, and PCNA being the pivotal down-stream mediators.

**Conclusions:**

Increased S100A15 expression and decreased DNA methylation of its gene promoter region were associated with high metastasis potential and poor outcome in lung adenocarcinoma, probably through triggering CTNNB1 -centered pathways.

## INTRODUCTION

The majority of non-small cell lung cancer patients present with advanced disease at diagnosis, and those diagnosed with early stage disease often recur and experience metastatic disease eventually [1]. Metastasis represents the primary cause of death from lung cancer, is responsible for 90% of all morbidity, and involves several stages, including invasion, angiogenesis, and migration. [2].

Human S100A15 (S100A7A; koebnerisin) is a novel member of the S100 family of EF-hand calcium-binding proteins and was recently identified in psoriasis, where it is significantly up-regulated in lesion skin. It is an antimicrobial protein, and regulated by E. coli through Toll-like receptor 4 [3]. Both human S100A7 (psoriasin) and S100A15 transcripts are elevated in ER/PR negative breast cancers; however, hS100A15 protein is detected in all cancer specimens, while hS100A7 protein is sporadically expressed [4, 5]. C-Fos transcriptionally controls S100A15 expression in keratinocytes, subsequently leading to CD4 T-cell recruitment to the skin, thereby promoting epidermal squamous cell carcinoma that is likely induced by CD4^+^ T-cell-derived IL-22 [6]. In our previous whole-genome gene expression study, we found that nuclear accumulation of S100A15 were increased in advanced stage NSCLC patients [1], but the potential of S100A15 as a determinant of treatment outcomes, and the underlying mechanisms by which S100A15 would be involved in proliferation, migration, and invasion of lung cancer have not yet been understood [1, 7]. DNA methylation changes induced in oncogenes and tumor suppressor genes are of key importance in the pathogenesis of lung cancer [8, 9]. Recent results indicate that changes in S100 protein expression may depend on the extent of DNA methylation in the S100 gene promoter regions [10]. The role of DNA methylation patterns of the S100A15 promoter region in metastasis potential of lung cancer has not yet been determined [11].

We retrospectively examined S100A15 protein expression, as determined by immunohistochemistry (IHC) stain, in a cohort of 178 subjects with NSCLC diagnosed through bronchoscopy-guided biopsy, and correlated it with their clinical characteristics, including tumor stage (TNM staging), treatment response, and overall survival (1- and 3-year). DNA methylation levels of 5 CpG sites over the S100A15 gene promoter region and 1 CpG site over exon 1 of the S100A15 gene were measured in 51 DNA samples extracted from the formalin fixed paraffin-embedded (FFPE) tissues of lung adenocarcinoma. Furthermore, S100A15 expression and its gene promoter DNA methylation levels in lung cancer cell lines were measured, and the effect of S100A15 on metastatic potential and down-stream mediators of lung adenocarcinoma was tested *in vitro*.

## RESULTS

### S100A15 nuclear accumulation was increased in lung adenocarcinoma patients with distant metastasis, and related to poor survival in those receiving first line target therapy

S100A15 IHC nuclear stain were performed and evaluated in 178 pathologically-diagnosed lung cancer patients, including 115 cases (64.6%) of adenocarcinoma, 50 cases (28.1%) of squamous cell carcinoma, and 13 cases (7.3%) of small cell carcinoma. The demographic and clinical data of these subjects are presented in Table 1. Stepwise forward multivariate Cox regression analysis showed that advanced tumor stage (HR 3.149, 95%CI 1.719-5.761, p<0.001), first line use of cisplatin-based chemotherapy (HR 0.323, 95%CI 0.203-0.513, p<0.001), and first-line use of target therapy with erlotinib or gefitinib (HR 0.608, 95%CI 0.373-0.992, p=0.046) were independent factors predicting one-year all-cause mortality. Furthermore, advanced tumor stage (HR 2.188, 95%CI 1.074-4.457, p=0.031), N2/3 (HR 3.343/5.946, 95%CI 1.871-5.975/3.107-11.879, p<0.001), M1 (HR 2.095, 95%CI 1.381-3.179, p=0.001), ECOG PS (HR 1.182, 95%CI 1.022-1.367, p=0.025), first line use of cisplatin-based chemotherapy (HR 0.492, 95% CI 0.333-0.728, p<0.001), and second line use of pemetrexed chemotherapy (HR 0.214, 95% CI 0.064-0.712, p=0.012) are independent factors predicting three-year all-cause mortality.

**Table 1:** Baseline and clinical characteristics of all the lung cancer patients

	All Lung cancerpatients, N=178	Early stage (I-IIIA),Lung AC patients,N=24	Advanced stage(IIIB-IV), Lung ACpatients, N = 91	P value*
Age, years		66.7±8.3	63.8±12	0.268
Male, n (%)	115 (64.6)	11 (45.8)	49 (53.8)	0.48
Smoking history, n (%)	57 (34)	5 (20.9)	24 (26.4)	0.426
TNM stage, n (%)				
T1	15 (8.4)	5 (20.8)	5 (5.5)	<0.001
T2	67 (37.6)	16 (66.7)	31 (34.1)	
T3	17 (9.6)	1 (4.2)	8 (8.8)	
T4	79 (44.4)	2 (8.3)	47 (51.6)	
N0	33 (18.5)	14 (58.3)	10 (11)	<0.001
N1	23 (12.9)	1 (4.2)	15 (16.5)	
N2	74 (41.6)	9 (37.5)	37 (40.7)	
N3	48 (27)	0 (0)	29 (31.9)	
M0	62 (35)	24 (100)	14 (15.38)	<0.001
M1	116 (65)	0 (0)	77 (84.6)	
ECOG PS				0.003
0	36 (20.2)	11 (45.8)	14 (15.4)	
1	85 (47.8)	11 (45.8)	45 (49.5)	
2	27 (15.2)	0 (0)	18 (19.8)	
3	13 (7.3)	2 (8.3)	4 (4.4)	
4	17 (9.6)	0 (0)	10 (11)	

AC = adenocarcinoma; PS = performance status

*Compared between early stage and advanced stage lung AC patients

**Figure 1:**
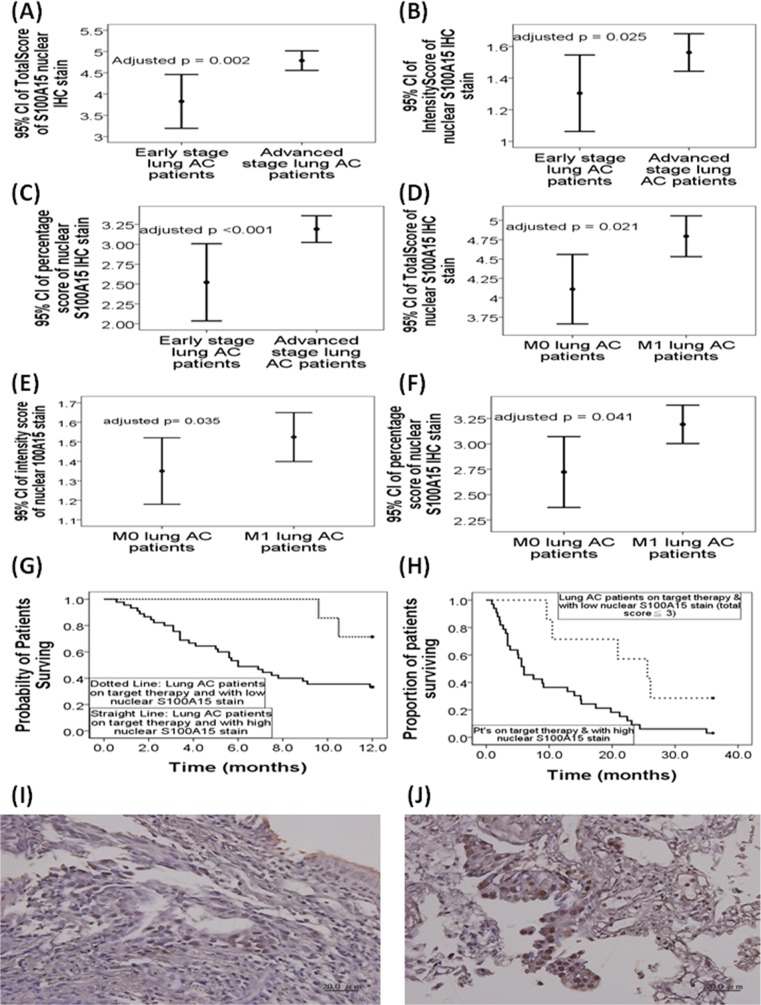
Immunohistochemistry (IHC) nuclear staining for S100A15 in patients with lung adeonocarcinoma (AC) In the 115 lung AC cases, **(A)** total score, **(B)** intensity score, and **(C)** percentage score of S100A15 nuclear stain were all significantly increased in patients with advanced tumor stage (stage IIIB and IV, n = 91) compared to that in those with early tumor stage (stage I - IIIA, n = 24). **(D)** Total score, **(E)** intensity score, and **(F)** percentage score of S100A15 nuclear stain were significantly increased in lung AC patients with distant metastasis (M1, n=77) as compared with that in those without distant metastasis (M0, n=38). Kaplan-Meier survival analysis showed significantly reduced **(G)** one-year overall survival (p=0.024 by log-rank test) and **(H)** three-year overall survival (p = 0.049 by the log-rank test) in the 33 AC patients receiving first line target therapy and harboring high nuclear expressions of S100A15 (total score < 3), compared with that in the 7 AC patients receiving first line target therapy and harboring low nuclear expressions of S100A15 (total score < 3). Immunohistochemistry analysis of S100A15 in paraffin embedded sections of **(I)** histologically proven AC, stage 1B, with nuclear percentage score 2 and nuclear intensity score 2, and **(J)** AC, stage 4, with nuclear nuclear percentage score 3 and intensity score 3.

In the 115 lung adenocarcinoma cases, total score (4.77±1.1 versus 3.75±1.48, adjusted p=0.002, 95%CI 0.126-0.538), intensity score (1.56±0.56 versus 1.29±0.55, adjusted p=0.025, 95%CI 0.015-0.209), and percentage score (3.18±0.8 versus 2.46±1.14, adjusted p<0.001, 95%CI 2.42-7.876) of S100A15 nuclear stain were all significantly increased in patients with advanced stage tumor (stage IIIB and IV, n = 91) compared with that in those with early stage tumor (stage I - IIIA, n = 24, Figure 1A-C). Likewise, total score (4.79±1.14 versus 4.05±1.34, adjusted p=0.012, 95%CI 0.154-1.217), intensity score (1.52±0.57 versus 1.35±0.53, adjusted p=0.035, 95%CI 0.017-0.47), and percentage score (3.18±0.81 versus 2.68±1.04, adjusted p=0.041, 95%CI 0.017-0.797) of S100A15 nuclear stain were significantly increased in lung adenocarcinoma patients with distant metastasis (M1, n=77) as compared with that in those without distant metastasis (M0, n=38, Figure 1D-F). Furthermore, Kaplan-Meier survival analysis showed significantly reduced one-year (p=0.024 by the log-rank test; median survival 6 months, Figure 1G) and three-year overall survival (p = 0.049 by the log-rank test; median survival 6 months, Figure 1H) in the 45 adenocarcinoma patients receiving first line target therapy and harboring high nuclear expressions of S100A15 (total score ≧ 4), compared with one-year median survival of 11.4 months and three-year median survival of 25.6 months in the 7 adenocarcinoma patients receiving first line target therapy and harboring low nuclear expressions of S100A15 (total score ≦ 3). There was no significant difference in S100A15 nuclear stain between early stage and advanced stage tumor or between distant metastasis and no distant metastasis in the other two pathological groups: small cell and squamous cell carcinoma. Figure 1I and 1J show representative pictures of IHC stain for S100A15 and its percentage / intensity score in patients with stage IB and stage IV lung adenocarcinoma, respectively.

### Decreased DNA methylation over -423/−412/−248 CpG sites of the S100A15 gene promoter region in lung adenocarcinoma patients with distant metastasis

**Figure 2:**
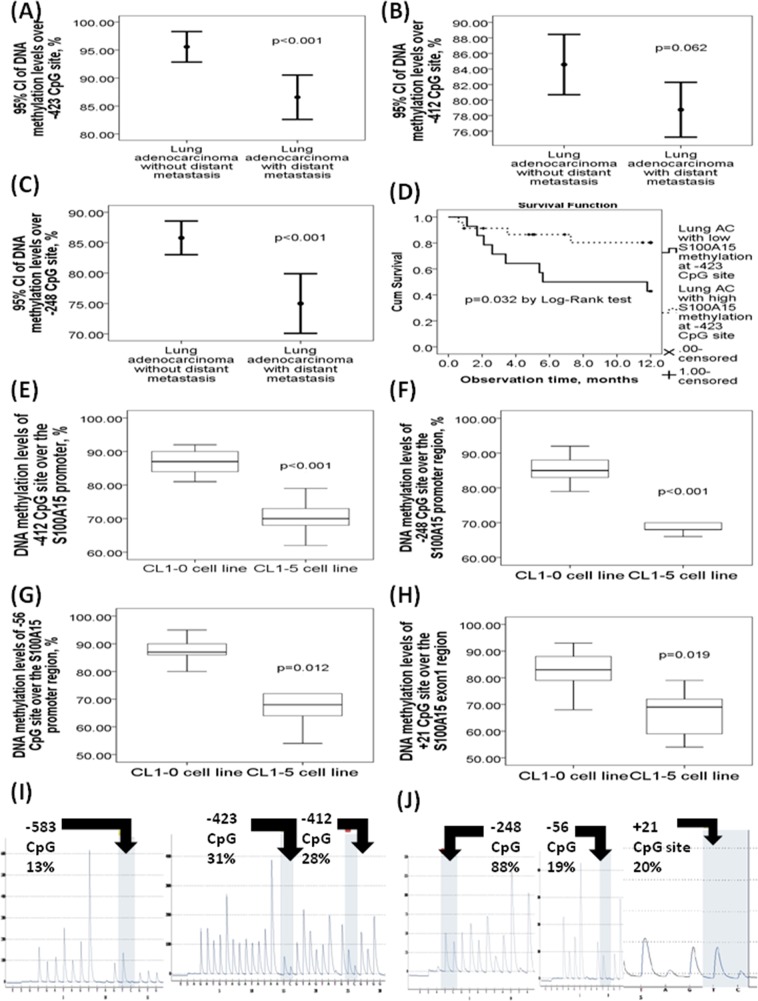
DNA methylation levels of the six CpG sites in the S100A15 gene promoter and exon 1 regions with respect to the clinical lung adenocarcinoma (AC) specimens and lung cancer cell lines DNA methylation levels of **(A)** -423 CpG site, **(B)** -412, and **(C)** -248 CpG sites were all decreased in 37 lung AC patients with distant metastasis versus 14 lung AC patients without distant metastasis. **(D)** Poorer one-year overall survival in lung AC patients with low DNA methylation levels (80%) over -423 CpG site of the S100A15 promoter as compared with those with high methylation levels. DNA methylation levels over **(E)** -412 CpG, **(F)** -248, and **(G)** -56 CpG sites of the S100A15 promoter, as well as **(H)** + 21 CpG site of the S100A15 exon 1 region were all decreased in CL1-5 cell line with high metastasis property versus that in the CL1-0 cell line with low metastasis property. **(I)** Representative pyrograms of the -583 CpG, -423 CpG, and -412 CpG sites in the S100A15 promoter region. **(J)** Representative pyrogrms of the -248, -56, and +21 CpG sites of the S100A15 gene.

To investigate the role of S100A15 gene promoter DNA methylation in the development of distant metastasis in lung AC patients, DNA methylation levels were measured by pyrosequencing in DNA samples extracted from the FFPE tissues of 37 adenocarcinoma patients with distant metastasis (M1), and 14 adenocarcinoma patients without distant metastasis (M0). DNA methylation levels of -423 CpG (86.57±11.89% vs. 95.57±4.72%, adjusted p<0.001, Figure 2A), -412 CpG (78.76±10.58% vs. 84.57±6.72%, adjusted p=0.062, Figure 2B), and -248 CpG (75±11.33% vs. 85.79±4.79%, adjusted p<0.001, Figure 2C) sites over the S100A15 gene promoter region were all significantly decreased in 37 AC patients with distant metastasis as compared with that in 14 AC patients without distant metastasis. Additionally, AC patients with low DNA methylation (80%) over -423 CpG site of the S100A15 gene promoter had significantly lower 1-year overall survival as compared with those with high DNA methylation (7.55±1.22 vs. 10.32±0.78 months, p=0.032 by Log-rank test, Figure 2D). Furthermore, DNA methylation levels over -412 CpG sites were significantly decreased in adenocarcinoma patients with a high percentage (>55%) of S100A15 nuclear stain positive cells as compared with that in those with a low percentage of S100A15 nuclear stain positive cells (80.7±15.8 vs. 90.9±10.8, p=0.043).

### Decreased DNA methylation levels over -412/−248/−56 CpG sites of the S100A15 gene promoter region and increased S100A15 gene/protein expression in lung cancer cell lines with high metastasis property

DNA methylation levels of the -412 (71±5.2% vs. 87.55±0.98%, p value < 0.001, Figure 2E), -248 (69.78±5.45 vs. 85.22±3.89%, p<0.001, Figure 2F), and -56 (69.88±12.46 vs. 86.66±5.66%, p=0.012, Figure 2G) CpG sites in the S100A15 gene promoter region, as well as the +21 CpG site (67±8.63 vs. 80.45±11.23%, p=0.019, Figure 2H) in the S100A15 exon1 region, were significantly decreased in CL1-5 cell line as compared with that in CL1-0. Representative pyrograms showed the percentage of methylation over the 6 CpG sites (Figure 2I and 2J).

**Figure 3:**
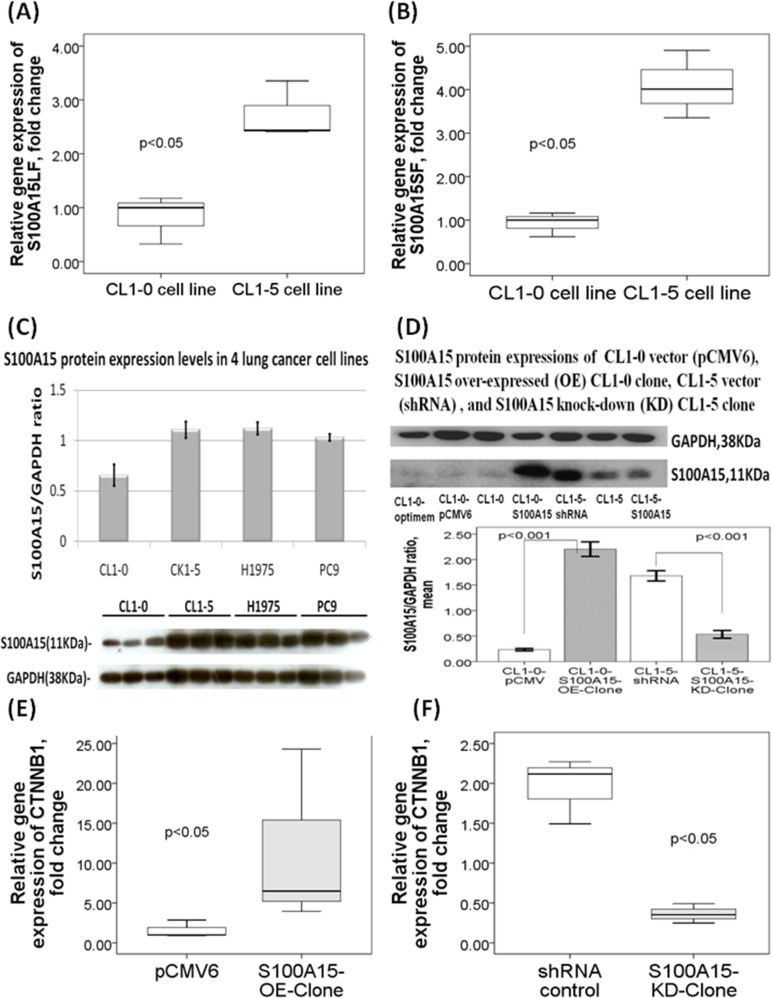
S100A15 gene and protein expressions in lung adenocarcinoma (AC) cell lines with high metastasis versus low metastasis properties Quantitative RT-PCR validation showed increased gene expressions of both **(A)** S100A15 long form (LF) and **(B)** S100A15 short form (SF) transcripts in CL1-5 cell line as compared with that in CL1-0 cell line. **(C)** Western blot results showed that S100A15 protein expression was significantly increased in the 3 lung cancer cell lines with high metastasis potential as compared to CL1-0 cell line (all p values < 0.05). **(D)** Western blot showed increased and decreased S100A15 protein expressions in S100A15 over-expressing CL1-0 cells and S100A15 knock-down CL1-5 cells, respectively. All experiments were performed independently for at least three times.

RNA-sequencing analysis showed that S100A15 gene expression was significantly increased in the three lung adenocarcinoma cell lines (CL1-5, PC9, H1975) with high metastasis potential as compared to that in CL1-0 lung adenocarcinoma cell line with low metastasis potential (all p values < 0.05). Real time quantitative RT-PCR analysis showed that gene expressions of both S100A15 splice variants long form (LF) and S100A15 short form (SF) were significantly increased in CL1-5 cell line as compared with that in CL1-0 cell line (both p values <0.05, Figure 3A and 3B) [4]. Likewise, Western blot analysis showed that S100A15 protein expression was significantly increased in the three lung cancer cell lines with high metastasis potential (S100A15/GAPDH ratio: CL1-5, 1.11L0.08, p=0.001; PC9, 1.03±0.03, p=0.002; H1975, 1.12±0.06, p<0.001) as compared with that in CL1-0 cell line (0.66±0.11, Figure 3C).

### Ectopic S100A15 expression increased cell proliferation, migration, and invasion

**Figure 4:**
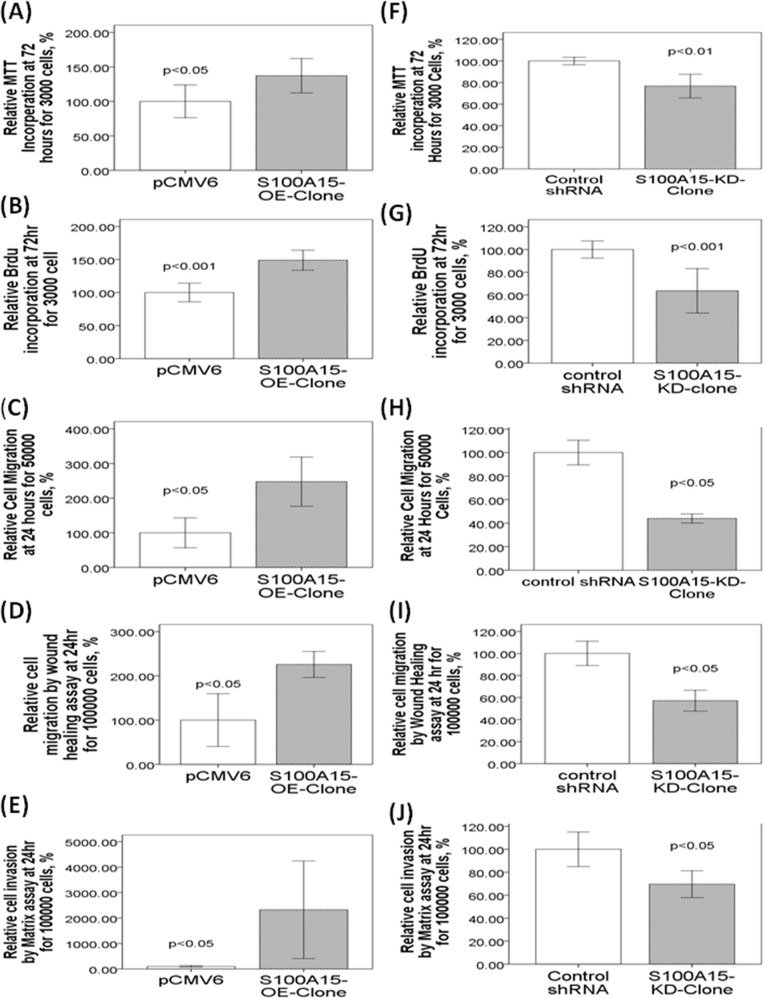
Over-expression and knock-down of S100A15 in CL1-0 and CL1-5 lung cancer cell lines, respectively, lead to changes in proliferative, migration, and invasive properties Enhanced S100A15 expression increased proliferation of CL1-0 cells. The cell proliferations of the control and S100A15-overexpressed CL1-0 cells were determined by **(A)** MTT and **(B)** BrdU incorporation analysis after 72 h of incubation. **(D)** Enhanced S100A15 expression increased migration of CL1-0 cells, as determined by **(C)** trans-well and **(D)** wound healing analysis after 24 h of incubation, and **(E)** increased invasion of CL1-0 cells. Knockdown of S100A15 decreased proliferation of CL1-5 cells, as determined by **(F)** MTT and **(G)** BrdU assays. Knock-down of S100A15 decreased migration of CL1-5 cells as determined by **(H)** trans-well and **(I)** wound healing analysis, and **(J)** decreased invasion of CL1-5 cells. The migratory and invasive cells were quantified by fluorescence dye staining. All experiments were performed independently at least three times.

To further investigate the role of S100A15, CL1-0 cells were transfected with S100A15 cDNA. Immunoblot analysis reveals that S100A15 was over-expressed in CL1-0 cells (S100A15/GAPDH ratio: CL1-0-S100A15-OE-clone 2.2±0.12 vs. CL1-0-pCMV6 0.23±0.02, p<0.001, Figure 3D). Over-expression of S100A15 significantly increased CL1-0 cell proliferation, as determined by MTT assay (relative change to control pCMV6 plasmid transfected CL1-0 cells: 137.22 ± 23.78%, P=0.014) (Figure 4A) and BrdU assay (relative change to control pCMV6 plasmid transfected CL1-0 cells: 138 ± 29.4%, p=0.038) (Figure 4B). In addition, the migration of S100A15-overexpressing CL1-0 cells was significantly increased compared with that of the control cells, as determined by trans-well migration assay (relative change to control pCMV6 plasmid transfected CL1-0 cells: 247.68 ± 28.6%, P<0.05) (Figure 4C) and wound healing assay (relative change to control pCMV6 plasmid transfected CL1-0 cells: 225.67±25.35%, p<0.05) (Figure 4D). The invasion of S100A15-overexpressing CL1-0 cells was significantly increased compared with that of the control cells as determined by matrix invasion assay (relative change to control pCMV6 plasmid transfected CL1-0 cells: 2327.16±1663.12%, p<0.05) (Figure 4E).

### Knockdown of S100A15 decreased cell proliferation, migration, and invasion

To investigate whether decreased S100A15 expression was associated with lung adenocarcinoma progression, S100A15 expression was reduced by shRNA transfection in CL1-5 cells. The S100A15 shRNA transfection showed inhibition of S100A15 protein expression in the stable CL1-5 clone (S100A15/GAPDH ratio, CL1-5-S100A15-KD-clone 0.53±0.07 vs. CL1-5-shRNA 1.68±0.09, p<0.001, Figure 3D). Knockdown of S100A15 significantly decreased CL1-5 cell proliferation, as determined by MTT assays (relative change to control shRNA plasmid-transfected CL1-5 cells: 76.7±15.5%, p=0.001) (Figure 4F) and BrdU assay (relative change to control shRNA plasmid-transfected CL1-5 cells: 63.6 ± 23.4%, p<0.001) (Figure 4G). In addition, the migration of S100A15-knockdown CL1-5 cells was significantly decreased compared with that in the control cells as determined by transwell migration assay (relative change to control shRNA plasmid-transfected CL1-5 cells: 43.91±3.32%, p<0.05) (Figure 4H) and wound healing assay (relative change to control shRNA plasmid-transfected CL1-5 cell: 57.15±8.25%, p<0.05) (Figure 4I). The invasion of S100A15-knockdown CL1-5 cells was significantly decreased compared with that of the control cells as determined by matrix invasion assay (relative change to control shRNA plasmid-transfected CL1-5 cells: 69.58±10.15%, p<0.01) (Figure 4J).

### Identification of S100A15-regulated genes by NGS

**Figure 5:**
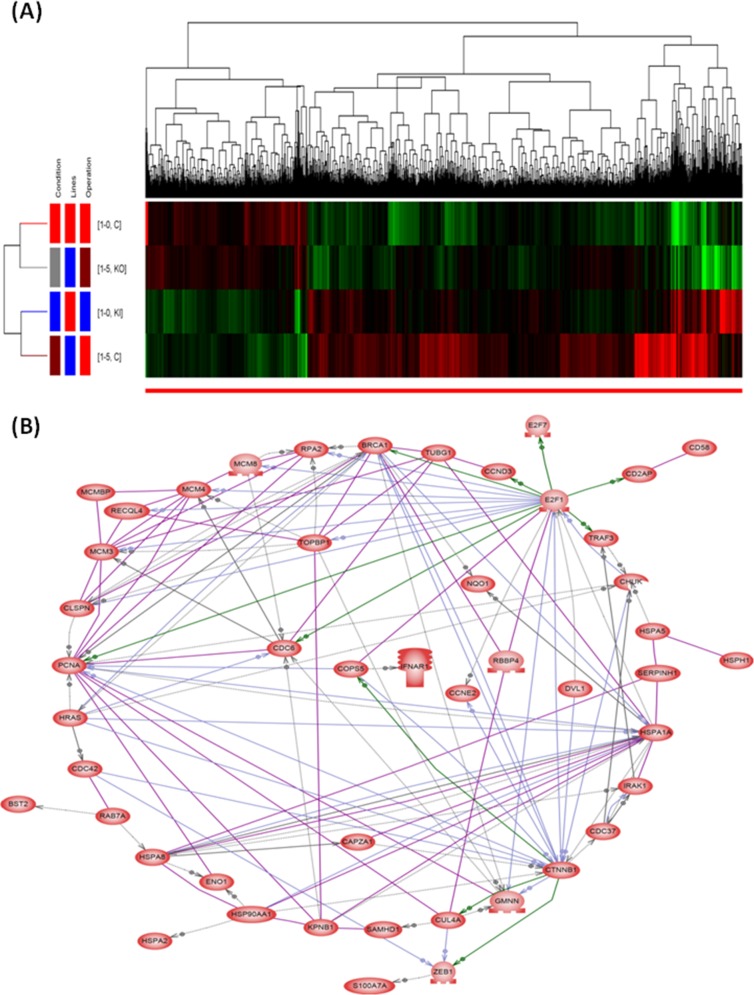
Hierarchical clustering dendrograms and direct interaction network of the significantly regulated genes by S100A15 **(A)** The red blocks represent under-expressed genes and the green blocks represent over-expressed genes. Totally, 518 differentially expressed genes were up-regulated and 1378 down-regulated in CL1-5 and S100A15 over-expressing CL1-0 as compared with CL1-0 and S100A15 knock-down CL1-5 lung AC cell lines. **(B)** The seed genes enriched by the sub-network analysis among the 518 genes up-regulated by S100A15.

Differentially expressed genes (DEG) between CL1-0 and S100A15-overexpressing CL1-0, and between CL1-5 and S100A15-knockdown CL1-5 cells were calculated from the raw reads using the DESeq method. Unsupervised hierarchical clustering identified 518 DEGs up-regulated and 1378 DEGs down-regulated in both S100A15-overexpressing CL1-0 and CL1-5 as compared with that in both CL1-0 and S100A15-knockdown CL1-5 cells (Figure 5A). Among the 518 DEGs, we further investigated gene functions and categorized according to specific biological processes by using the GO enrichment analysis. Abundantly enriched signaling pathways include gene sets involved in NOTCH signaling, cell proliferation, protein kinase binding, methyltransferase activity, and ubiquitin-dependent protein degradation (Supplementary Table 1). To better understand the biological context of the changes in lung cancer cell lines affected by S100A15, the 518 DEGs that were potentially induced by S100A15 were analyzed using Ariadne's pathway buildup for direct interaction and enriched by the sub-network enrichment analysis to identify putative expression networks and their corresponding regulators. The enriched sub-networks consisting of a set of single seed genes with target genes associated to the seed among these DEGs are presented in a circular pathway layout (Figure 5B). Forty six sub-networks altered between S100A15 over-expression and knockdown were built around the seed of CTNNB1 (β-catenin). Overall, our data reveal an important physiological role for S100-A15 in comprehending the proliferative, metastatic, and invasive properties of lung adenocarcinoma which may be orchestrated by CTNNB1 with the involvement of ZEB1, CDC42, HSP90AA1, PCNA and BST2. Using real time quantitative RT-PCT method, we demonstrated that CTNNB1 gene expression of the S100A15-overexpressing CL1-0 cells was significantly increased as compared with that of the control pCMV6 cells (fold change 11.57±11.08 vs. 1.59±1.1, p<0.05, Figure 3E), while that of the S100A15-knockdown CL1-5 cells was significantly decreased as compared with that of the control shRNA cells (fold change 0.36±0.12 vs. 1.96±0.41, p<0.05, Figure 3F).

## DISCUSSION

Recently, several S100A family members have been recognized as a critical regulator of the metastatic process and epithelial-mesenchymal transition occurrence in several human cancers. Among them, S100A4 and S100A6 have been reported to be epigenetically up-regulated in nasopharyngeal and gastric cancer by DNA hypomethylation over their gene promoter regions, while S100A2 and S100A10 down-regulated in head/neck and pituitary cancer by DNA hypermethylation [12, 13]. Our data provide evidence that S100A15 aggravates metastatic progressing in lung adenocarcinoma *in vivo* and *in vitro* through DNA hypomethylation over its gene promoter region, and CTNNB1-centered down-stream mediators.

Several S100 family genes can differently modulate tumor cells, tumor environment, and tumor cell migration to influence epithelial carcinogenesis. Both the S100 family genes containing CpG islands in their promoter regions, including S100A11, S100A2, S100A6, and S100A10, as well as relatively CpG-poor genes (S100A4), can be silenced by DNA methylation [10]. DNA methylation at promoter-associated CpG islands or individual CpG site involves association of methyl-binding domain proteins, histone deacetylases, and inhibitory histone modifications, and rebuilds chromatin to a tightly packed, transcriptionally inactive form, abrogating the binding of a transcription factor and RNA polymerase 2 [14]. For the first time, we found that S100A15 promoter hypomethylation at the three CpG sites and its enhanced expression were both associated with a higher metastasis potential and poorer outcome in lung adenocarcinoma patients. Moreover, we verified this phenomenon in lung adenocarcinoma cell lines with high versus low metastasis properties. In line with our findings, DNA hypomethylation and enhanced gene expression of S100A4 can increase invasive ability and promote metastasis in nasopharyngeal, laryngeal, and endometrial carcinoma [12, 15, 16]. Likewise, increased S100A6 expression and its DNA hypomethylation are associated with poor prognosis in gastric cancer [13]. S100A6 and S100A10 demonstrated tumor-specific hypermethylation in medulloblastoma primary tumors and cell lines, which was associated with their transcriptional silencing, while decreased S100A10 expression associated with increased promoter CpG methylation was noted in primary human pituitary tumors [17, 18]. Because nuclear accumulation of S100A15 was evidenced by IHC stain in the lung adenocarcinoma patients with distant metastasis, we speculate that its nuclear translocation from beneath the plasma membrane region is the first step to exert its down-stream oncogenic activities. Further investigation is required to clarify the relationship between DNA hypomethylation of the S100A15 gene promoter and its nuclear translocation.

The lack of clinical association with S100A15 in the other two pathological types (squamous cell and small cell carcinoma) of lung cancer in the present study could be attributed to several reasons. First, the interaction between epidermal growth factor receptor and S100A family can promote angiogenesis and metastasis in a variety of cancers, whereas the percentage of EGFR mutations is relatively small in these 2 types of lung cancers [19, 20]. Second, some S100A family members contribute to progression of squamous cell carcinomas, while others maintain the differentiated state of epithelium and contribute to a less invasive tumor type [21–24]. Although relative strong expression of nuclear S100A15 was found in squamous cell carcinoma, its biological function in this type of lung cancer remains to be determined. Third, little expression of the S100A family is found in a variety of small cell cancers [25, 26]. S100A15 may not play a pivotal role in small cell lung cancer.

S100A15 binds directly to HER2 and regulates MMP2 to contribute to cell proliferation and invasion of breast cancer, respectively [27]. With tumor progression, S100A7 translocates into the nucleus, where the psoriasin—Jab1 complex transactivates tumor-promoting AP-1 targets and oncogenic COP9 signalosome signaling, while loses the cytoplasmic function as a negative regulator of β-catenin mediated oncogenic c-Myc activity. On the other hand, S100A15 downstream signaling that could be important for tumor cell survival remains to be largely unknown [7, 28]. For the first time, our NGS data identified 518 DEGs up-regulated by S100A15 and 1378 DEGs down-regulated by S100A15, with the former mapped to 46 sub-network seed genes. Among them, CTNNB1, ZEB1, CDC42, HSP90AA1, BST2, PCNA, and E2F1 have all been shown to promote lung cancer progression, while SAMHD1, HRAS, and NQO1 serve as tumor suppressor genes [29–33].

In summary, this study provides compelling evidence that S100A15 promote tumor progression in lung adenocarcinoma. S100A15 may exert its oncogenic function, initiated by DNA hypomethylation over its gene promoter region and mediated by CTNNB1-centered down-stream genes. Both enhanced S100A15 expression and its gene promoter DNA hypomethylation can serve as biomarkers predicting high metastasis potential and poor outcome in lung adenocarcinoma patients. Further investigation on S100A15 functions and its epigenetic regulations may provide a potential treatment strategy for lung cancer.

## MATERIALS AND METHODS

### Subjects and clinical phenotypes in lung cancer

FFPE tissue sections of lung tumor had been obtained from a cohort of 178 patients undergoing bronchoscopy-guided biopsy for diagnosis of lung tumor at the broncho-scope room of Kaohsiung Chang Gung Memorial Hospital (KCGMH) during 2007 through 2009. Tissue procurement was approved by the Institutional review board of the CGMH (certificate number: 102-0911B). Final pathological diagnoses included AC, squamous cell carcinoma, small cell carcinoma, and large cell carcinoma.

### IHC staining and its evaluation for S100A15

FFPE sections were deparaffinized, and antigen retrieval was carried out as previously described [1]. The tissue sections were then incubated for 1 h at room temperature with anti-human S100 calcium binding protein A15 (anti-hS100A15; 1:5000; a gift from Stuart H. Yuspa, Laboratory of Cancer Biology and Genetics Center for Cancer Research, National Cancer Institute, USA) overnight, followed by 30 min-incubation with goat anti-mouse IgG conjugated to a horseradish peroxidase-labeled polymer (Envision+ System; DakoCytomation, Carpinteria, California, USA).

Each slide was evaluated for S100A15 immunostaining using a semi-quantitative scoring system for both staining intensity and the percentage of positive tumor/pulmonary epithelial cells as described previously [1]. IHC staining overview was performed by two independent trained readers, who were blinded to the clinical outcome, tumor stage, and histopathology. The tissue sections were scored by manually counting ≥ 500 cells based on the percentage of immunostained cells (0–10% = 0; 10–30% = 1; 30–50% = 2; 50–70% = 3 and 70–100% = 4) and staining intensity (negative = 0; mild = 1; moderate = 2; and intense = 3). A total score of nuclear staining was obtained by adding the scores of percentage positivity and staining intensity.

### Measurement of DNA methylation levels over S100A15 gene promoter region by bisulfite pyro-sequencing method

Genomic DNA was isolated from the FFPE tissues and cancer cell lines using a genomic DNA purification kit (Puregene). Four regions of the S100A15 promoter element (GenBank accession number, NM_176823.3; Gene ID, 338324 (S100A7A)), including six CpG cites (−583, -423, -412, -248, -56, and +21 from transcription start site; chromosome 1: 153415941, 153416101, 153416112, 153416276, 153416468, and 153416544, based on UCSC genome browser GRCh38/hg38 Assembly) were amplified. Bisulfate treatment was performed using EpiTect 96 Bisulfite Kit (Qiagen) and PCR amplification was performed using PyroMark PCR Kit (Qiagen), as described previously [34]. The PCR condition was 45 cycles of 95°C for 20 s, 50°C for 20 s, and 72°C for 20 s, followed by 72°C for 5 min. Primer sequences used for PCR amplification and pyro-sequencing for the 5 regions were listed in Table 2. The biotin-labeled PCR product was captured by Streptavidin-Sepharose HP (Amersham Pharmacia). Quantitation of cytosine methylation was done using the PyroMark Q24 system (Qiagen). The amount of C relative to the sum of the amounts of C and T at each CpG site was calculated as percentage.

**Table 2:** PCR amplification, pyrosequencing, and RT-PCR primers used in measuring DNA methylation and mRNA levels of the S100A15 gene and its promoter regions

Primers	Sequences
Region 1 for -583 CpG site	
Forward PCR Primer	5′-GATTTATAGTATAGGGGATAAGAGTAAGGA
Biotinylated Reverse PCR Primer	5′-TCCCAAATTTACTCAACTAATAAACACAAC
Forward Sequencing Primer	5′-GGGGATAAGAGTAAGGAT
Region 2 for -423/−412 CpG site	
Forward PCR Primer	5′-GGGTTGAGGAGGATGTAAGAGTAGT
Biotinylated Reverse PCR Primer	5′-CTCCCCCAATACTTCCACACATTT
Forward Sequencing Primer	5′-AAGAGTAGTAGGTGTTTT
Region 3 for -248 CpG site	
Biotinylated Forward PCR Primer	5′-TGTTTATTAGTTGAGTAAATTTGGGAATAT
Reverse PCR Primer	5′-CAAACTCCCAAAAACAACATAATCTTATT
Reverse Sequencing Primer	5′-ACACATTTATTATAACAACCTATT
Region 4 for -56 CpG site	
Forward PCR Primer	5′-ATGTTGTTTTTGGGAGTTTGAAAT
Biotinylated Reverse PCR Primer	5′-AACACACCCAAAAAACTATACTC
Forward Sequencing Primer	5′-ATTTTTTGATTTGTTATTATATATG
Region 5 for +21 CpG site	
Forward PCR Primer	5′-AGGTTGAGTTTTATAAAGGATTGTTT
Biotinylated Reverse PCR Primer	5′-CCCAAACTTCTAAAACTTTATCCACAA
Forward Sequencing Primer	5′-ATAAAGGATTGTTTTTTGTTTAAAT
S100A15 LF RT-PCR	
hS100A15LF-Forward	5′-ACGTCACTCCTGTCTCTCTTTGCT
hS100A15LF-Reverse	5′-TGATGAATCAACCCATTTCCTGGG
S100A15 SF real-time RT-PCR	
hS100A15SF-Forward	5′-CAAGTTCCTTCTGCTCCATCTTAG
hS100A15SF-Reverse	5′-AGCCTTCAGGAAATAAAGACAATC
GAPDH real-time RT-PCR	
Forward	5′-GAAGAGCCAAGGACAGGTAC
Reverse	5′-CAACTTCATCCACGTTCACC

### *In vitro* experimental procedures

#### Lung cancer cell line culture

The human lung AC cell line CL1 was obtained from a 64-year-old man with a poorly differentiated adenocarcinoma [35]. The CL1-0 (low metastasis potential), CL1-5 (high metastasis), PC9 (Deletion of Exon 19 of EGFR, high metastasis), and H1975 (L858R+T790M mutations of EGFR, high metastasis) cells were all provided by Dr. Pan-Chyr Yang (Department of Internal Medicine, National Taiwan University Hospital, Taipei, Taiwan, Republic of China) and were cultured at 37°C with 5% CO2 in RPMI 1640 media that was supplemented with 10% fetal bovine serum (FBS)1 (Invitrogen, Gaithersburg, MD) and 2 g/liter NaHCO3. The four cancer cells were grown in serum- containing media until the cell density reached 70–80% confluence in the culture dish. After incubation, the total number of viable and dead cells in the four cancer cells was determined using the trypan blue dye exclusion assay. The percentage of cell viability was determined to be > 98%.

### *In vitro* gene knockdown and over-expression

The lipofectamine 2000 transfection reagent was used to achieve gene silencing of S100A15 by transfection with the shRNA, which was performed according to the manufacturer's instructions (Santa Cruz Biotechnology, Santa Cruz, CA). Half a microgram of S100A15 shRNA or half a microgram of scrambled control shRNA with 6 μl of shRNA transfection reagent (3 × 10^5^ cells/well) was used for each shRNA transfection. Knockdown of S100A15 in CL1-5 was performed using lentiviruses, which were produced by co-transfecting HEK293T with pLKO-AS3-S100A15 and packaging plasmids (pCMV6). The stable clones were established by puromycin selection. The efficacy of the S100A15 shRNA plasmid was assessed by flow cytometry and Western blot methods. S100A15 gene over-expression was achieved through transfection with a commercial virus vector using the TurboFect transfection reagent according to the manufacturer's instructions (OriGene Technologies, Inc., Rockville, MD). For each well of transfection, 4 μg of virus DNA or empty vector control with 12 μl of TurboFect transfection reagent was added to 100 μl of serum-free medium. The solution was mixed gently and overlaid onto the cells in complete medium supplemented with 10% FBS for further study. For S100A15 overexpression, CL1-0 cells were transfected with pCMV6 or pCMV6-S100A15 plasmid by Lipofectamine for 8 hours.

### Western blot analysis

Fifty μg of proteins isolated from the lung cancer cell lines were separated on a 10% SDS-PAGE, transferred on a nitrocellulose membrane (Millipore, Germany), and probed with primary antibody (S100A15 1:2000; a gift from Stuart H. Yuspa, Laboratory of Cancer Biology and Genetics Center for Cancer Research, National Cancer Institute, USA) or (GAPDH 1:15000; Epitomics) followed by secondary antibody (anti-rabbit, 1:5000, Millipore). Detection was performed by ImmunStar AP Chemiluminescent Kit (Bio-Rad Lab., Germany).

### Measurement of S100A15 mRNA gene expressions by quantitative real-time reverse transcription (RT)-polymerase chain reaction (PCR) method

Total RNA from whole cell lysates of the lung adenocarcinoma cell lines was isolated by RNA Extraction RiboPureTM-Blood (Ambion), and converted to single-stranded cDNA using a cDNA archive kit (Applied Biosystems) followed by the amplification of the S100A15 transcript by using Taqman probe and specific primers (Table 2). *GAPDH* was used as the internal control. The PCR reaction was performed at 94°C for 10 minutes, followed by amplification (95°C for 10 seconds, 60°C for 30 seconds), and cooling (40°C for 30 seconds), for 30 cycles. The PCR products were subjected to 1% agarose gel electrophoresis and photographed. Relative expression levels were calculated using the ΔΔCq method with the median value for the control group as the calibrator.

### Cell proliferation assays

3-[4,5-dimethylthiazol-2-yl]-2,5-diphenyltetrazolium bromide (thiazolyl blue, MTT) proliferation analyses were performed in a 96-well plate with the cells seeded at a concentration of 3 × 10^3^ cells per well. MTT (10 mg/ml, 2 μL) was added to each well and incubated for another 3 h at 37°C. The cells were lysed with 100 μl of dimethyl sulfoxide. The cell growth of each group was monitored after 72 h, using Microplate photometers by measuring the absorbance at 590 nm (Biotium, USA). A Bromodeoxyuridine (BrdU) cell proliferation assay was performed according to the manufacturer's instructions (BIOVISION, USA). A total of 3 × 10^3^ cells were seeded in each well of a 96-well plate, and cell growth within each group was monitored at 72 h.

### Cell wound healing assay

Cell migration was examined with the commercial Millicell® Hanging Cell Culture inserts single and preloaded inserts (MILLIPORE, Switzerland). Cells were seeded on the insert for 12 h, and the inserts were removed. Photographs were taken at 0 h and 24 h at the same position in the cell-free gap insert with 100× magnification. Adobe PHOTOSHOP CS6 and MRI Wound Healing Tool were used to calculate the area occupied by migrating cells.

### Transwell migration and matrix invasion assays

Cell migration was examined in a membrane migration culture system with Millicell® Hanging Cell Culture inserts single and preloaded inserts systems (MILLIPORE, Switzerland) with 8-μm pores. The upper wells contained 1 × 10^5^ cells seeded in serum-free medium, and the lower chambers were filled with complete medium supplemented with 10% FBS to induce cell migration. After the cells were incubated at 37°C for 24 h, the membranes were fixed with methanol, the cells were stained with a Giemsa stain, and the number of cells that migrated through the membrane to the lower side was determined. The cells traversing the filter to the lower chamber were counted at 200× magnification in ten fields per filter. In preparation for the invasion assay, Millicell® Hanging Cell Culture inserts single and preloaded inserts systems (MILLIPORE, Switzerland) containing 8-μm pores were coated with 2 mg/ml QCM ECMatrix Cell Invasion basement membrane matrix (MILLIPORE, USA & Canada), following the same steps as those of the trans-well migration assay.

### Next generation sequencing (NGS) for mRNA-Sequencing analysis

Total mRNA was isolated from four clones of the lung cancer cell lines (CL1-0, CL1-5, CL1-0 with S100A15 OE, and CL1-5 with S100A15 KD) using TRIzol reagent (Invitrogen). Total RNA (15 μg) for each sample was used for purifying the poly(A)-containing mRNA molecules, RNA amplification, and synthesis of double stranded cDNAs that will be ligated to adapters, following the Illumina (San Diego, CA) TruSeq RNA Sample Prep guidelines. Multiplexed samples were sequenced at 100bp length on an in-house Illumina MiSeq instrument as described previously [36]. Briefly, sequences called by the Illumina pipeline were mapped to the reference genome and annotated using Strand NGS 2.1 (Strand Life Sciences, Bangalore, Karnataka, India). All expression dataset has been deposited in the NCBI Gene Expression Omnibus (GEO) with accession number GSE86344. The results from NGS analysis were analyzed by Mann–Whitney unpaired test or two-way analysis of variance and a Benjamini and Hochberg false discovery rate multiple gene correction was applied. To better understand the biological context of the changes, data were analyzed by Ariadne's Pathway Studio (Elsevier B.V., Amsterdam, Netherlands) to build up for direct interaction and subnetwork enrichment analysis.

### Statistical analysis

Data were expressed as the mean ± SEM. One-way analysis of variance (ANOVA) were be used for comparing mean values of more than two experimental groups. Student t-test, non-parametric U-test or Wilcoxon Signed-Ranks test was used for comparing mean or median values of two experimental groups, where appropriate. Categorical variables were analyzed using Chi-square test. Statistical modeling for prognostic factors was done, incorporating age, gender, co-morbidities, smoking status, ECOG performance status, disease stage, treatment strategies, and histology, one at a time, into the main Cox model. The Kaplan-Meier method was used to estimate overall survival. Multiple linear regression analysis was used to minimize the effects of confounding factors on the subgroup of continuous variables, and to provide adjusted p values with 95% confidence interval (CI). A p-value of less than 0.05 is considered statistically significant. All analyses were performed using the SPSS 15.0 software (SPSS Corp., Chicago).

## SUPPLEMENTARY MATERIALS TABLES




