# Supplementary material for: Increased S100A15 expression and decreased DNA methylation of its gene promoter are involved in high metastasis potential and poor outcome of lung adenocarcinoma
Source: Oncotarget. 2017 Apr 24;8(28):45710–24. doi: 10.18632/oncotarget.17391 (PMC5542220; doi:10.18632/oncotarget.17391)
Supplement: Supplementary file 1 [file oncotarget-08-45710-s001.pdf]

Increased S100A15 expression and decreased DNA methylation of its gene promoter are involved in high metastasis potential and poor outcome of lung adenocarcinoma

SUPPLEMENTARY MATERIALS

SUPPLEMENTARY TABLE

Supplementary Table 1: Functional categories of the 518 differentially expressed genes (DEG) up-regulated in both CL1-5 and S100A15 over-expressing (OE) CL1-0, and down-regulated in both CL1-0 and S100-A15 knock-down (KD) CL1-5 lung cancer cell lines.

See Supplementary File 1
