# Supplementary material for: Increased S100A15 expression and decreased DNA methylation of its gene promoter are involved in high metastasis potential and poor outcome of lung adenocarcinoma
Source: Oncotarget. 2017 Apr 24;8(28):45710–24. doi: 10.18632/oncotarget.17391 (PMC5542220; doi:10.18632/oncotarget.17391)
Supplement: Supplementary file 2 [file oncotarget-08-45710-s002.docx]

**Supplementary Table 1. Functional categories of the 518 differentially expressed genes (DEG) up-regulated in both CL1-5 and S100A15 over-expressing (OE) CL1-0, and down-regulated in both CL1-0 and S100-A15 knock-down (KD) CL1-5 lung cancer cell lines.**

| **Signaling pathway** | **Gene Symbol** | **S100A15 manipulation** | | | | **Description** |
| --- | --- | --- | --- | --- | --- | --- |
|  |  | **CL1-0** | | **CL1-5** | |  |
|  |  | Control | OE | Control | KD |  |
| NOTCH Signaling | CTNNB1 | 0.763 | 0.977 | 1.249 | 1.023 | catenin (cadherin-associated protein), beta 1, 88kDa |
|  | RBBP4 | 0.830 | 1.070 | 1.495 | 0.934 | retinoblastoma binding protein 4 |
|  | HMGN2 | 0.748 | 1.338 | 1.442 | 0.744 | high mobility group nucleosomal binding domain 2 |
|  | RBBP7 | 0.633 | 1.027 | 1.205 | 0.974 | retinoblastoma binding protein 7 |
|  | CHMP1A | 0.963 | 1.330 | 1.039 | 0.871 | charged multivesicular body protein 1A |
|  | DMAP1 | 0.828 | 1.150 | 1.152 | 0.870 | DNA methyltransferase 1 associated protein 1 |
|  | POLE3 | 0.925 | 1.464 | 1.082 | 0.786 | polymerase (DNA directed), epsilon 3, accessory subunit |
|  | NAP1L4 | 0.660 | 1.058 | 1.309 | 0.945 | nucleosome assembly protein 1-like 4 |
|  | H2BFS | 0.264 | 2.825 | 2.773 | 0.361 | H2B histone family, member S (pseudogene) |
|  | BAZ1A | 0.987 | 1.587 | 1.013 | 0.796 | bromodomain adjacent to zinc finger domain, 1A |
|  | SUPT16H | 0.952 | 1.398 | 1.050 | 0.871 | suppressor of Ty 16 homolog (S. cerevisiae) |
|  | BANF1 | 0.922 | 1.217 | 1.063 | 0.941 | barrier to autointegration factor 1 |
|  | HELLS | 0.916 | 1.686 | 1.092 | 0.785 | helicase, lymphoid-specific |
|  | MBD2 | 0.683 | 1.111 | 1.099 | 0.910 | methyl-CpG binding domain protein 2 |
|  | KAT2A | 0.913 | 1.162 | 1.096 | 0.877 | K(lysine) acetyltransferase 2A |
|  | SMARCD2 | 0.916 | 1.186 | 1.092 | 0.822 | SWI/SNF related, matrix associated, actin dependent regulator of chromatin, subfamily d, member 2 |
| Cell Proliferation | CTNNB1 | - |  |  |  |  |
|  | TXNRD1 | 0.910 | 1.276 | 1.098 | 0.781 | thioredoxin reductase 1 |
|  | TFDP1 | 0.789 | 1.267 | 1.734 | 0.718 | transcription factor Dp-1 |
|  | E2F1 | 0.840 | 1.190 | 2.001 | 0.750 | E2F transcription factor 1 |
|  | ZEB1 | 1.027 | 0.833 | 0.974 | 1.183 | zinc finger E-box binding homeobox 1 |
|  | HDGF | 0.942 | 1.313 | 1.062 | 0.834 | hepatoma-derived growth factor |
|  | E4F1 | 0.928 | 1.293 | 1.077 | 0.844 | E4F transcription factor 1 |
|  | RBBP7 | - |  |  |  |  |
|  | NUDC | 0.738 | 1.616 | 1.288 | 0.776 | nuclear distribution C homolog (A. nidulans) |
|  | PCNA | 0.592 | 1.627 | 1.188 | 0.842 | proliferating cell nuclear antigen |
|  | UBE2V2 | 0.753 | 1.012 | 1.184 | 0.989 | ubiquitin-conjugating enzyme E2 variant 2 |
|  | KAT2A | - |  |  |  |  |
|  | RRN3 | 0.883 | 1.246 | 1.133 | 0.666 | RRN3 RNA polymerase I transcription factor homolog (S. cerevisiae) |
|  | ZNF259 | 0.834 | 1.362 | 1.199 | 0.754 | zinc finger protein 259 |
|  | HRAS | 0.847 | 1.181 | 1.312 | 0.598 | v-Ha-ras Harvey rat sarcoma viral oncogene homolog |
|  | GNB1 | 0.892 | 1.157 | 1.121 | 0.844 | guanine nucleotide binding protein (G protein), beta polypeptide 1 |
|  | BST2 | 0.426 | 0.703 | 35.452 | 1.423 | bone marrow stromal cell antigen 2 |
|  | PRMT5 | 0.804 | 1.278 | 1.208 | 0.828 | protein arginine methyltransferase 5 |
|  | TACC1 | 0.798 | 1.151 | 1.227 | 0.869 | transforming, acidic coiled-coil containing protein 1 |
| Protein Kinase Binding | CTNNB1 | - |  |  |  |  |
|  | ATP1A1 | 0.655 | 0.856 | 1.292 | 1.168 | ATPase, Na+/K+ transporting, alpha 1 polypeptide |
|  | PGAM1 | 0.606 | 1.091 | 1.391 | 0.917 | phosphoglycerate mutase 1 |
|  | HSP90AA1 | 0.619 | 2.869 | 1.213 | 0.824 | heat shock protein 90kDa alpha (cytosolic), class A member 1 |
|  | ACTB | 0.866 | 1.306 | 1.154 | 0.722 | actin, beta |
|  | CDC42 | 0.692 | 1.076 | 1.151 | 0.929 | cell division cycle 42 (GTP binding protein, 25kDa) |
|  | DVL1 | 0.830 | 1.137 | 1.170 | 0.880 | dishevelled, dsh homolog |
|  | TRAF3 | 0.864 | 1.158 | 1.175 | 0.665 | TNF receptor-associated factor 3 |
|  | CCND3 | 0.671 | 1.077 | 1.985 | 0.928 | cyclin D3 |
|  | CCNE2 | 0.637 | 1.198 | 3.828 | 0.835 | cyclin E2 |
|  | FGFR1OP | 0.734 | 1.084 | 1.133 | 0.923 | FGFR1 oncogene partner |
|  | YWHAZ | 0.609 | 0.847 | 1.304 | 1.181 | tyrosine 3-monooxygenase/tryptophan 5-monooxygenase activation protein, zeta polypeptide |
|  | CDC37 | 0.957 | 1.515 | 1.044 | 0.902 | cell division cycle 37 homolog |
|  | PPME1 | 0.769 | 1.131 | 1.585 | 0.884 | protein phosphatase methylesterase 1 |
| Methyltransferase Activity | TYMS | 0.784 | 1.216 | 1.319 | 0.822 | thymidylate synthetase |
|  | DMAP1 | - |  |  |  |  |
|  | PRMT7 | 0.948 | 1.417 | 1.055 | 0.795 | protein arginine methyltransferase 7 |
|  | PRMT1 | 0.964 | 1.392 | 1.037 | 0.847 | protein arginine methyltransferase 1 |
|  | PRMT5 | - |  |  |  |  |
|  | ICMT | 0.810 | 1.477 | 1.234 | 0.644 | isoprenylcysteine carboxyl methyltransferase |
|  | NTMT1 | 0.999 | 1.452 | 1.001 | 0.859 | N-terminal Xaa-Pro-Lys N-methyltransferase 1 |
|  | SETD6 | 0.626 | 1.428 | 1.163 | 0.860 | SET domain containing 6 |
|  | TYW3 | 0.840 | 1.363 | 1.132 | 0.883 | tRNA-yW synthesizing protein 3 homolog |
|  | SMYD5 | 0.802 | 1.090 | 1.119 | 0.917 | SMYD family member 5 |
|  | METTL16 | 0.835 | 1.196 | 1.158 | 0.864 | methyltransferase like 16 |
| Ubiquitin-Dependent Protein Degradation | UBE2V2 | 0.753 | 1.012 | 1.184 | 0.989 | ubiquitin-conjugating enzyme E2 variant 2 |
|  | UBE2N | 0.934 | 1.573 | 1.071 | 0.915 | ubiquitin-conjugating enzyme E2N |
|  | PSMC1 | 0.865 | 1.234 | 1.157 | 0.846 | proteasome (prosome, macropain) 26S subunit, ATPase, 1 |
|  | PSMC3 | 0.865 | 1.283 | 1.156 | 0.762 | proteasome (prosome, macropain) 26S subunit, ATPase, 3 |
|  | UBE2D3 | 0.788 | 0.991 | 1.159 | 1.009 | ubiquitin-conjugating enzyme E2D 3 |
|  | PSMD5 | 0.891 | 1.137 | 1.122 | 0.770 | proteasome (prosome, macropain) 26S subunit, non-ATPase, 5 |
|  | PSMB10 | 0.794 | 1.260 | 2.089 | 0.667 | proteasome (prosome, macropain) subunit, beta type, 10 |
|  | PSMA7 | 0.771 | 1.276 | 1.146 | 0.873 | proteasome (prosome, macropain) subunit, alpha type, 7 |
|  | PSMD12 | 0.884 | 1.528 | 1.094 | 0.914 | proteasome (prosome, macropain) 26S subunit, non-ATPase, 12 |
|  | PSMA5 | 0.637 | 1.080 | 1.152 | 0.926 | proteasome (prosome, macropain) subunit, alpha type, 5 |
